# Association of preoperative state anxiety and early postoperative recovery in living kidney donors undergoing nephrectomy: A prospective observational study

**DOI:** 10.1016/j.jatmed.2026.07.001

**Published:** 2026-08-11

**Authors:** Maham Javed Chohan, Syed Jawad Ali Bukhari, Zargham Waheed, Munawal Javaid, Zaeema Ahmad, Sara Sarfraz, Amnah Khalid

**Affiliations:** Department of Anaesthesiology, Bahria International (SAFARI) Hospital, Rawalpindi 46000, Pakistan

**Keywords:** Kidney donors, Nephrectomy, Anxiety, Postoperative recovery, Postoperative pain

## Abstract

**Background:**

Preoperative anxiety may influence perioperative physiological responses, early postoperative recovery, postoperative pain, and patient satisfaction in living kidney donors. This study evaluated the association between preoperative state anxiety and early postoperative recovery outcomes in adults undergoing elective living kidney nephrectomy.

**Methods:**

In this prospective observational study, 100 consecutive living kidney donors undergoing elective donor nephrectomy at Bahria International (SAFARI) Hospital, Rawalpindi, Pakistan, from January 2024 to December 2025, were enrolled. The primary endpoint was post-anaesthesia care unit (PACU) stay duration. Secondary endpoints included extubation time, time to achieve Modified Aldrete Score ≥9, postoperative pain, analgesic consumption, patient satisfaction, postoperative nausea and vomiting (PONV), and postoperative length of stay. Preoperative state anxiety was assessed on the day before surgery using the State-Trait Anxiety Inventory-State Scale (STAI-S) in English and Urdu. Skewed outcomes were summarized as median and interquartile range [IQR], while the Kruskal-Wallis test was used for anxiety group comparison. STAI-S was approximately normally distributed and is reported as mean and standard deviation. Multiple linear regression was performed to identify predictors of PACU stay adjusted for prespecified confounders.

**Results:**

The mean STAI-S score was 43.14 ± 11.97, with n = 44 (44%) donors categorized as low, n = 33 (33%) as moderate, and n = 23 (23%) as high anxiety. Higher anxiety was associated with longer extubation time, delayed Aldrete recovery, longer PACU stay, greater pain at 1 h and 6 h, lower patient satisfaction, and longer hospital stay. In multivariable analysis, higher preoperative anxiety scores were associated with longer PACU stay duration (*β* = 0.768, p < 0.001), explaining 55.8% of the variance (R² = 0.570).

**Conclusions:**

Higher preoperative state anxiety scores were associated with lower early postoperative recovery scores, greater early postoperative pain, longer hospital stay, and lower patient satisfaction among living kidney donors.

## Introduction

Living kidney donors constitute a distinct surgical population because they are generally healthy individuals undergoing major surgery primarily for the recipient’s benefit, without direct therapeutic benefit to themselves. Ensuring optimal recovery and minimizing avoidable perioperative morbidity in this population is therefore ethically, clinically, and operationally important. While physical outcomes and donor safety have long been emphasized, donor-specific data linking preoperative anxiety with standardized early recovery outcomes remain limited.^1^, ^2^, ^3^ A previous study involving living kidney donors reported that higher preoperative anxiety was associated with delayed return of spontaneous respiration, delayed extubation, and prolonged discharge time from the post-anesthesia care unit (PACU).^1^ However, donor-specific evidence remains limited, particularly from South Asian transplant settings where donor demographics, sociocultural expectations, perioperative counselling, and institutional recovery pathways may differ.^1^ Donor anxiety may also differ from routine surgical anxiety because it can include concerns about recipient outcome, family expectations, voluntariness of donation, future health, and return to normal function.^1^, ^2^, ^3^

Preoperative anxiety is highly prevalent among patients waiting for elective surgery, with reported prevalence estimates ranging from 60% to 80% of different surgical patients, though donor-specific prevalence may differ.^4^ This prevalence, combined with evidence linking anxiety to poorer outcomes, substantiates the rationale for focusing on anxiety in donor nephrectomy. The high prevalence of preoperative anxiety also indicates that donor populations cannot be assumed to be free of anxiety simply because they are healthy volunteers.^5^ This psychological burden is not confined to major operations or high-risk population; even healthy individuals scheduled for surgery may experience elevated anxiety. The relevance of this in the donor nephrectomy setting is underscored by prior donor-nephrectomy findings, where living renal donors with higher anxiety scores exhibited delayed emergence from anesthesia. These data suggest that anxiety may not only reflect emotional distress but can also manifest physiologically in delayed recovery kinetics.^1^

Mechanistically, anxiety triggers a cascade of psychophysiological responses known to influence surgical outcomes. Increased perioperative sympathetic activity may increase pain perception, and contribute to delayed functional recovery.^6^ These mechanisms are of particular interest in living donors, whose postoperative course is otherwise typically expected to be uncomplicated. Furthermore, living donors have a unique psychosocial context involving emotional issues, family pressures, and concerns for the recipient, all of which may contribute to increased perioperative anxiety.^2^

The anaesthetic plan adopted in donor nephrectomy also has a strong bearing on early postoperative recovery outcomes. The anesthesia component, including choice of agents, fluid, and analgesic strategies, plays a critical role in shaping postoperative recovery.^7^ Anesthetic technique may influence early recovery after donor nephrectomy.^8^ Furthermore, literature shows that balanced general anesthesia was associated with a substantially lower risk of major adverse cardiac events, suggesting potential organ-protective effects and enhanced recovery outcomes in a renal patient context.^9^ Bridging these two domains, preoperative anxiety and anaesthetic technique together create a compelling rationale to examine whether the donor’s psychological state modifies postoperative recovery. High anxiety may increase anaesthetic requirements,^10^ whereas reductions in perioperative anxiety may improve early postoperative recovery.^11^ Furthermore, the use of validated recovery metrics provides an integrated measure of physical and emotional recovery rather than isolated physiological endpoints.^12^

In this prospective observational study, living kidney donors undergoing elective donor nephrectomy were recruited. Building on previous donor-nephrectomy evidence, this study aimed to externally validate and extend the observed association between preoperative state anxiety and early postoperative recovery in a Pakistani transplant setting. The primary objective was to assess the association between preoperative state anxiety and PACU stay duration in living kidney donors undergoing elective donor nephrectomy. The secondary objectives were to assess the association between preoperative anxiety and extubation time, time to Modified Aldrete Score ≥ 9, postoperative pain, analgesic consumption, patient satisfaction, postoperative nausea and vomiting, and postoperative hospital stay.

## Method

### Study design and approval

The prospective observational study was conducted in the Department of Anaesthesiology at Bahria International (SAFARI) Hospital, Rawalpindi, Pakistan, a tertiary care referral center for renal transplantation, from February 2024 to December 2025. The study population comprised healthy adult volunteers undergoing elective donor nephrectomy as part of a living kidney donation. Ethical approval for the study was obtained from the Institutional Ethical Review Committee (IERC) of Bahria International (SAFARI) Hospital, Rawalpindi (REF NO: IERC/BIH-RWP/2024/003), and written informed consent was obtained from all participants before enrollment. The study was conducted and reported in accordance with the Strengthening the Reporting of Observational Studies in Epidemiology (STROBE) reporting guideline for observational studies and the principles of the Declaration of Helsinki.

### Study participants

Participants were recruited consecutively if they were adult living kidney donors aged 18–65 years, classified as American Society of Anesthesiologists (ASA) physical status I or II, medically cleared for elective donor nephrectomy after institutional transplant evaluation, approved through the Punjab Human Organ Transplantation Authority (PHOTA) authorization process, and scheduled to undergo surgery under balanced general anesthesia. In this study, “healthy donors” referred to donor candidates who had fulfilled institutional and PHOTA donor-suitability requirements before enrolment; therefore, candidates with impaired renal function, uncontrolled hypertension, diabetes mellitus, or clinically significant metabolic or systemic disease were not eligible for donation and were excluded from enrollment. Study-specific exclusion criteria were documented psychiatric illness, current use of anxiolytic or psychotropic medication, inability to complete the State-Trait Anxiety Inventory-State Scale (STAI-S), refusal to participate, and known hypersensitivity to propofol or any other study-related anaesthetic drug.

### Sampling technique and sample size

The sample size was calculated for the primary endpoint, PACU stay duration, using a multiple linear regression framework. Assuming a moderate association between preoperative anxiety and early recovery outcomes based on previous donor-nephrectomy literature, an anticipated effect size of f² = 0.19, a two-sided alpha level of 0.05, 80% statistical power, and up to seven predictors in the adjusted model, the minimum required sample size was 83 participants. To allow for possible attrition, incomplete data, and to improve model stability, a target sample size of 100 donors was selected. Participants were recruited using consecutive non-probability sampling, whereby all eligible living kidney donors presenting during the study period were approached until the target sample size was achieved. The study was powered for the primary adjusted association with PACU stay duration and not for formal stratified subgroup analyses.

### Data collection

Following enrollment, participants underwent a detailed preoperative assessment the day before surgery. Demographic and clinical variables, including age, sex, body mass index (BMI), education level, smoking status, previous surgical history, and ASA grade, were recorded. Preoperative anxiety was assessed using the State-Trait Anxiety Inventory-State Scale (STAI-S), a validated psychometric tool measuring situational anxiety. Scores range from 20 to 80, with higher scores indicating greater state anxiety. The questionnaires were administered in a quiet preoperative area by trained staff to ensure participant comfort and comprehension.

### Anesthesia protocols

All participants were managed according to a standardized institutional anesthesia protocol. Standard intraoperative monitoring included continuous electrocardiography, non-invasive blood pressure, pulse oximetry, capnography, end-tidal anaesthetic gas monitoring, temperature monitoring, and urine output measurement. General anesthesia was induced intravenously with Propofol (2.5 mg/kg), Nalbuphine (0.1 mg/kg), and a neuromuscular blocker (Atracurium 0.6 mg/kg). Anesthesia was maintained with balanced general anesthesia technique with Oxygen, isoflurane, and Nitrous Oxide, titrated to maintain adequate depth of anesthesia based on clinical signs and hemodynamic parameters.

Intraoperative fluid therapy was guided by haemodynamic parameters, urine output, surgical losses, and institutional donor-nephrectomy practice, using balanced crystalloid solutions. Analgesia was standardized with intraoperative nalbuphine and postoperative opioid administration according to institutional protocol and patient pain scores. Antiemetic prophylaxis was administered according to institutional practice and patient requirement. At the end of surgery, inhalational agents were discontinued, residual neuromuscular blockade was reversed, and patients were extubated upon meeting standard clinical criteria, including adequate spontaneous ventilation, haemodynamic stability, protective airway reflexes, and appropriate response to verbal command.

### Outcome measures

Recovery parameters were observed from cessation of anaesthetic agents through the PACU phase. The primary endpoint was total PACU stay duration, defined as the time from arrival in the post-anesthesia care unit to fulfilment of discharge-readiness criteria. PACU stay was selected as the primary endpoint because it provides an integrated measure of early anaesthetic recovery and served as the dependent variable in the adjusted regression analysis. Secondary endpoints included time to extubation define as the time from discontinuation of anesthetic agents to tracheal extubation, time to achieve Modified Aldrete Score (≥9), postoperative pain at 1, 6, and 24 h using the Visual Analogue Scale (VAS), where 0 represented no pain, and 10 represented the worst possible pain, total 24h postoperative analgesic consumption recorded by drug type and dose. Other postoperative variables included the incidence of postoperative nausea and vomiting (PONV), length of hospital stay, and patient satisfaction, rated on a 10-point numeric scale. Importantly, the investigators collecting intra- and postoperative data were blinded to the patients’ preoperative anxiety scores to minimize observer bias.

### Ethical consideration

As the living donors were healthy volunteers undergoing non-therapeutic surgery, special emphasis was placed on the voluntary nature of the participation, confidentiality, and the absence of coercion. The participation of the donors did not influence the evaluation or the decision-making regarding the surgery. Data integrity was maintained by employing standardized forms for data collection and double data entry into a secure, password-protected database. Each participant was assigned a unique study identification number to preserve confidentiality. Daily verification of collected data was performed by the principal investigator, and discrepancies were resolved through review of source documents. All data were anonymized before analysis. The study was conducted in accordance with the international, local, and institutional guidelines. Participation was entirely voluntary, and all participants were informed of their right to withdraw at any time without any impact on their clinical care. All data were kept confidential and used exclusively for research purposes.

### Statistical analysis

Statistical analysis was carried out using SPSS version 27.0. Normality of continuous data was assessed using the Shapiro-Wilk test. Continuous variables were summarized as median (IQR) due to non-normality, while categorical variables were presented as frequencies and percentages. Baseline demographic and clinical characteristics were compared across low-, moderate-, and high-anxiety groups. Continuous variables were compared using the Kruskal–Wallis test, while categorical variables were compared using the chi-square test or Fisher’s exact test, as appropriate. Baseline variables showing significant between-group differences were included as additional covariates in the multivariable regression model. STAI-S was analyzed both as a continuous variable and as categorical anxiety groups (20–37 low anxiety, 38–44 moderate anxiety, and 45–80 high anxiety). These thresholds were used to facilitate clinical interpretation and comparison with previous literature; they were not applied as diagnostic cutoffs. For the primary adjusted analysis, STAI-S was retained as a continuous variable.^13^ Differences between the anxiety groups were analyzed with the Kruskal-Wallis test. When the Kruskal–Wallis test was significant, Bonferroni-adjusted post hoc pairwise comparisons were performed between anxiety categories. Adjusted *p* values were reported for low versus moderate, low versus high, and moderate versus high anxiety groups. Pairwise comparisons were not performed when the overall Kruskal–Wallis test was not significant. To explore adjusted associations with PACU stay, multivariable linear regression was performed. Assumptions of linear regression, including residual normality, homoscedasticity, and the absence of influential observations, were assessed. Exploratory interaction analyses were performed to assess whether the association between preoperative STAI-S score and PACU stay duration differed by age or gender. Interaction terms for STAI-S × age and STAI-S × gender were entered separately into the adjusted regression model to avoid overfitting. A two-sided *p* value less than 0.05 was considered statistically significant.

## Results

The baseline demographic and clinical characteristics of the study participants are summarized in Table 1. The median age of the donors was 37.00 (31.25–42.75) years, with the majority belonging to the 31–40 years age group, n = 44 (44.0%). In terms of gender distribution, females constituted a larger proportion, n = 59 (59%), while regarding marital status, the majority of participants were married, n = 73 (73%). The median body mass index (BMI) was 22.80 kg/m² (20.04–26.07), and most participants were ASA physical status I (77%). The highest proportion of respondents had attained secondary education, n = 39 (39%), while concerning employment status, unemployed were the largest employment category (n = 31, 31%), followed by students (n = 25, 25%), employed participants and housewives (n = 22, 22% each). Only a small proportion of participants reported a previous surgical history, n = 13 (13%), whereas n = 91 (91%) were non-smokers.

**Table 1:** Baseline demographics and clinical characteristics of the study population (n, %; Median (IQR)).

**Variable**	**Groups**	**Frequency (*****f*****)**	**Percentage (*****%*****)**
Age (years)	Median (IQR)	37.00 (31.25–42.75)	
Age category	18–30	19	19.0%
	31–40	44	44.0%
	> 40	37	37.0%
Gender	Male	41	41.0%
	Female	59	59.0%
Marital status	Unmarried	27	27.0%
	Married	73	73.0%
BMI (kg/m²)	Median (IQR)	22.80 (20.04–26.07)	
ASA Physical status	ASA I	77	77.0%
	ASA II	23	23.0%
Educational level	Primary	21	21.0%
	Secondary	39	39.0%
	Graduate	27	27.0%
	Postgraduate	13	13.0%
Employment status	Employed	22	22.0%
	Unemployed	31	31.0%
	Housewife	22	22.0%
	Student	25	25.0%
Surgical history	Yes	13	13.0%
	No	87	87.0%
Smoking history	Yes	9	9.0%
	No	91	91.0%

Data are presented as n (%) and median (interquartile range, IQR). n: number, BMI: Body Mass Index, ASA: American Society of Anesthesiologists.

Baseline characteristics were compared across anxiety categories (Table S1). Age, body mass index, sex, ASA physical status, education level, employment status, previous surgical history, and smoking history did not differ significantly among low-, moderate-, and high-anxiety groups. Marital status differed significantly across anxiety categories (χ² = 8.108, p = 0.017) and was therefore included as an additional covariate in the multivariable regression model.

### Reliability of the STAI-S scale

The STAI-S, consisting of 20 items, demonstrated a Cronbach’s alpha of 0.875, indicating good internal consistency of the STAI-S in this sample. This suggests that the items within the scale are highly correlated and reliably measure the same underlying construct of state anxiety. A Cronbach’s value above 0.8 reflects that the scale items are consistent and reliable, supporting internal consistency reliability in this sample.

#### State‐Trait Anxiety Inventory (STAI) score and anxiety level

Table 2 shows that the mean STAI-S score among participants was 43.14 ± 11.97, indicating that the average score fell within the moderate range, though scores were widely dispersed. Based on predefined cutoffs, n = 44 (44%) of participants was classified as having low anxiety, n = 33 (33%) moderate anxiety, and n = 23 (23%) experienced high anxiety levels. This distribution suggests 56.0% of donors had moderate-to-high preoperative anxiety.

**Table 2:** Distribution of STAI-S and anxiety category (n, %, mean ± SD).

**STAI-S categories**	**Frequency (*****f*****)**	**Percentage (*****%*****)**	**(Mean±SD)**
STAI-S Scores	-	-	43.14 ± 11.97
Low anxiety	44	44.0	-
Moderate anxiety	33	33.0	-
High anxiety	23	23.0	-
Total	100	100.0	-

Data are presented as n (%) and mean (SD). SD = Standard Deviation, STAI-S: State-Trait Anxiety Inventory-State Scale.

### Perioperative and early postoperative outcomes

Table 3 shows the intraoperative and early recovery outcomes. The median total dose of propofol used was 167.50 (IQR: 150.00–196.88) mg, indicating moderate variability in anesthetic requirements among participants. The median duration of anesthesia was 145.00 (IQR:136.00–158.00) min, suggesting moderate procedural length variability. The median extubation time was 7.00 (IQR: 6.00–7.00) min, reflecting efficient postoperative airway recovery. Similarly, the Aldrete recovery time had a median of 14.00 (IQR: 12.00–16.00) min, indicating that most patients achieved satisfactory recovery within a reasonable time frame. Notably, intraoperative complications were rare, occurring in only n = 4 (4%) of cases, while n = 96 (96%) of patients experienced uneventful intraoperative courses. Overall, these findings demonstrate stable intraoperative and recovery profiles, with minimal complications and efficient postoperative recovery times, suggesting effective anesthesia management across different anxiety categories.

**Table 3:** Perioperative and early postoperative outcomes (Median (IQR), n, %).

**Parameter**	**n, %; Median (IQR)**	
Total propofol used (mg)	167.50 (150.00–196.88)	
Anesthesia duration (min)	145.00 (136.00–158.00)	
Extubation time (min)	7.00 (6.00–7.00)	
Aldrete recovery time (min)	14.00 (12.00–16.00)	
Intraoperative complications (Yes/No)	4 (4.0%)/96 (96.0%)	
VAS Pain 1 h	4.00 (3.00–4.00)	
VAS Pain 6 h	3.00 (3.00–4.00)	
VAS Pain 24 h	2.00 (1.00–2.00)	
PONV (Yes/No)	2 (2.0%)/98 (98.0%)	
PACU Stay (min)	60.00 (56.00–67.00)	
Total opioid consumption in 24 h (mg)	100.50 (90.00–117.75)	
Patient satisfaction score	8.00 (7.00–8.00)	
Hospital stay (h)	40.00 (32.00–45.00)	

Data are presented as median (interquartile range, IQR).

The median VAS pain score at 1 h postoperatively was 4.00 (IQR: 3.00–4.00), indicating moderate pain immediately after surgery. Pain levels gradually declined over time, with median scores of 3.00 (IQR: 3.00–4.00) at 6 h and 2.00 (IQR: 1.00–2.00) at 24 h. The median total analgesic consumption within 24 h was 100.50 (IQR: 90.00–117.75) mg, while the incidence of PONV was very low, reported in only n = 2 (2%) of cases. The median PACU stay was 60.00 (IQR: 56.00–67.00) min, and the overall patients reported a median satisfaction score of 8.00 (7.00–8.00). The median hospital stay duration was 40.00 (IQR: 32.00–45.00) h.

#### Postoperative outcomes across anxiety categories (Kruskal-Wallis Test)

In Table 4, the Kruskal-Wallis test was performed, which demonstrated differences in various postoperative recovery outcomes across categories of anxiety (low, moderate, and high). Recovery outcomes differed significantly across anxiety categories. Compared with donors with low anxiety, those with moderate and high anxiety had progressively longer extubation times, longer times to achieve an Aldrete score ≥9, longer PACU stays, higher pain scores at 1 h, lower satisfaction scores, and longer postoperative hospital stays (all *p* < 0.001). Bonferroni-adjusted post hoc comparisons confirmed significant pairwise differences between low-, moderate-, and high-anxiety groups for extubation time, Aldrete recovery time, PACU stay duration, pain at 1 h, patient satisfaction, and hospital stay. Pain at 6 h also differed across groups (H = 8.327, *p* = 0.016), although the pattern was less marked, whereas neither total analgesic use over the first 24 h (H = 4.213, *p* = 0.122) nor pain at 24 h (H = 2.703, *p* = 0.259) showed significant between-group differences. Overall, higher preoperative anxiety was associated with less favorable immediate postoperative recovery.

**Table 4:** Postoperative outcomes across anxiety categories with Bonferroni-adjusted post hoc pairwise comparisons.

**Outcome**	**Low anxiety Median (IQR)**	**Moderate anxiety Median (IQR)**	**High anxiety Median (IQR)**	**Kruskal–Wallis H**	**Overall*****p*****value**	**Low vs Moderate adj*****p***	**Low vs High adj*****p***	**Moderate vs High adj*****p***
Total analgesic use in first 24 h (mg)	105.75 (94.50–124.50)	99.00 (88.50–106.50)	97.50 (88.50–115.50)	4.213	0.122	—	—	—
Time from end of anesthesia to extubation (min)	6.00 (5.00–6.00)	7.00 (7.00–7.00)	8.00 (8.00–8.50)	81.172	<0.001	<0.001	<0.001	0.001
Time to achieve Aldrete score ≥ 9 (min)	11.50 (11.00–13.00)	15.00 (14.00–16.00)	18.00 (17.00–18.00)	83.744	<0.001	<0.001	<0.001	0.001
Total PACU stay duration (min)	54.50 (47.50–57.00)	63.00 (62.00–65.00)	71.00 (68.00–80.50)	86.106	<0.001	<0.001	<0.001	0.001
Postoperative pain at 1 h, VAS 0–10	3.00 (3.00–3.00)	4.00 (4.00–4.00)	5.00 (5.00–5.00)	84.470	<0.001	<0.001	<0.001	<0.001
Postoperative pain at 6 h, VAS 0–10	3.00 (3.00–4.00)	3.00 (3.00–4.00)	3.00 (3.00–3.00)	8.327	0.016	0.020	1.000	0.086
Postoperative pain at 24 h, VAS 0–10	2.00 (1.00–2.00)	2.00 (1.00–2.00)	2.00 (2.00–2.00)	2.703	0.259	—	—	—
Patient satisfaction score	8.00 (8.00–9.00)	7.00 (7.00–7.00)	7.00 (6.00–7.00)	74.465	<0.001	<0.001	<0.001	0.017
Postoperative hospital stay (h)	32.00 (29.00–34.00)	42.00 (40.00–42.00)	54.00 (50.00–55.00)	85.830	<0.001	<0.001	<0.001	0.001

Data are presented as median (interquartile range, IQR). Kruskal–Wallis test was used to compare outcomes across low-, moderate-, and high-anxiety groups. For outcomes with significant Kruskal–Wallis tests, post hoc pairwise comparisons were performed using Bonferroni-adjusted pairwise tests. Adjusted p values are reported. Pairwise comparisons were not performed for outcomes with non-significant overall Kruskal–Wallis tests and are indicated by “—”. A two-sided *p* value < 0.05 was considered statistically significant. IQR, interquartile range; PACU, post-anesthesia care unit; VAS, visual analogue scale.

### Predictors of PACU stay duration

**Fig. 1:**
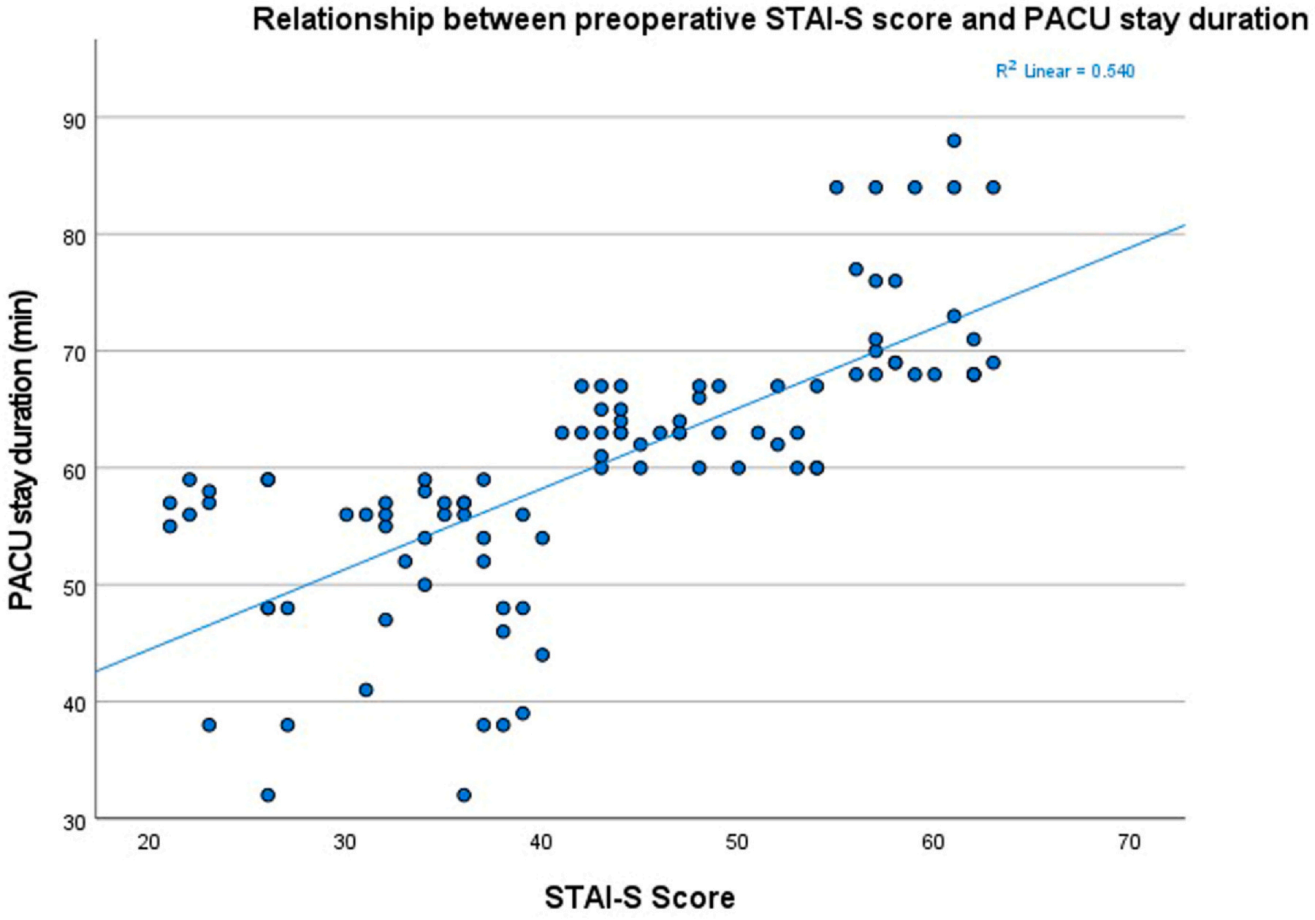
Scatter plot showing the relationship between preoperative STAI-S score and PACU stay duration. Each point represents one participant. The solid line represents the fitted linear regression line, demonstrating a positive association between preoperative state anxiety and PACU stay duration. PACU, post-anesthesia care unit; STAI-S, State-Trait Anxiety Inventory-State Scale.

In multivariable linear regression, the overall model for PACU stay duration was statistically significant (Table 5), explaining 57.0% of the variance in PACU stay duration (R = 0.755, R² = 0.570, adjusted R² = 0.538, F[7,92] = 17.453, *p* < 0.001). After adjustment for age, gender, marital status, body mass index, ASA physical status, and duration of anesthesia, higher STAI-S total score remained significantly associated with longer PACU stay (B = 0.708, SE = 0.067, standardized *β* = 0.756, 95% CI: 0.575–0.841; *p* < 0.001). This indicates that each 1-point increase in STAI-S score was associated with an estimated 0.708-minute increase in PACU stay duration. None of the other covariates were significantly associated with PACU stay, including age (*p* = 0.069), gender (*p* = 0.673), marital status (*p* = 0.101), body mass index (*p* = 0.421), ASA physical status (*p* = 0.195), and duration of anesthesia (*p* = 0.794). Variance inflation factors ranged from 1.043 to 2.362, indicating no evidence of problematic multicollinearity. The positive linear relationship between STAI-S score and PACU stay duration is illustrated in Fig. 1, which shows that higher preoperative anxiety scores were associated with longer PACU stay. In exploratory interaction analyses, neither the STAI-S × age interaction (B = 0.000, SE = 0.010, *β* = 0.004, p = 0.961) nor the STAI-S × gender interaction (B = −0.169, SE = 0.135, *β* = −0.143, p = 0.215) was significantly associated with PACU stay duration—suggesting the anxiety–recovery association is consistent across age and gender subgroups (Table S2).

**Table 5:** Multiple linear regression predicting Post-Anesthesia Care Unit (PACU) Stay Duration after adjustment for baseline imbalance.

**Predictor**	**Unstandardized B**	**SE**	**Standardized β**	**95% CI for B**	**p value**	**VIF**
STAI-S total score	0.708	0.067	0.756	0.575–0.841	<0.001	1.101
Age	0.334	0.181	0.193	−0.026–0.694	0.069	2.349
Gender	0.681	1.610	0.030	−2.516–3.878	0.673	1.080
Marital status	−4.374	2.638	−0.174	−9.612–0.865	0.101	2.362
Body mass index	0.141	0.174	0.057	−0.206–0.487	0.421	1.076
ASA physical status	−2.453	1.878	−0.093	−6.183–1.276	0.195	1.076
Anesthesia duration (min)	−0.011	0.042	−0.018	−0.095–0.073	0.794	1.043

Model statistics: R = 0.755, R² = 0.570, adjusted R² = 0.538, F(7,92) = 17.453, *p* < 0.001. Dependent variable: Total PACU stay duration, minutes. *ASA,* American Society of Anesthesiologists; CI, confidence interval; PACU, post-anesthesia care unit; SE, standard error; STAI-S, State-Trait Anxiety Inventory-State Scale; VIF, variance inflation factor.

## Discussion

The prospective observational study evaluated the association between preoperative state anxiety and early postoperative recovery outcomes in living kidney donors undergoing donor nephrectomy under balanced general anesthesia. The findings demonstrated that higher preoperative state anxiety was significantly associated with several early postoperative recovery parameters. Participants with higher anxiety scores experienced significantly longer extubation times, delayed achievement of an Aldrete score ≥9, prolonged post-anesthesia care unit (PACU) stay and length of hospital stay, greater pain intensity at 1 h postoperatively, and lower patient satisfaction. Multivariable analysis confirmed that the STAI-S score was the only significant independent predictor of PACU stay after adjustment for age, gender, body mass index, marital status, ASA physical status, and duration of anesthesia.

These findings are consistent with a previous study of Turksal et al.*,* which reported associations between higher anxiety and delayed anaesthetic recovery, increased postoperative pain, greater analgesic requirement, and lower satisfaction in living kidney donors.^1^ The present study extends this evidence by evaluating a larger donor cohort from a Pakistani transplant centre, providing external validation in a different sociocultural and healthcare setting, assessing anxiety both categorically and continuously, and applying multivariable adjustment for clinical covariates and baseline imbalance. By also including Modified Aldrete recovery time and postoperative hospital stay, this study provides a broader assessment of early recovery and strengthens the donor-specific evidence base association.^1^, ^3^, ^14^

The findings are clinically relevant because living kidney donors are medically selected healthy individuals undergoing major surgery primarily for recipient benefit rather than for direct therapeutic gain. In this context, even modest prolongation of extubation time, PACU stay, hospital stay, pain, or dissatisfaction has ethical and operational significance. The mean STAI-S score indicated moderate state anxiety overall, with more than half of donors exhibiting moderate-to-high anxiety, supporting the view that healthy donor status should not be interpreted as absence of psychological vulnerability. Our findings are consistent with donor-specific evidence from Turksal et al.^1^ and broader perioperative literature showing that preoperative anxiety is associated with delayed recovery, greater postoperative discomfort, and poorer postoperative experience.^3^, ^10^, ^14^

Anxiety may influence early recovery through several psychophysiological pathways. Sympathetic activation, heightened cortisol and catecholamine release, increased vigilance to bodily sensations, and negative pain appraisal may increase anaesthetic requirement, delay emergence, and amplify early nociceptive sensitivity. The temporal pattern of pain findings is clinically informative. Pain differed clearly at 1 h, was weaker at 6 h, and no longer differed significantly at 24 h. This pattern may reflect heightened anticipatory distress, sympathetic arousal, and increased vigilance to bodily sensations during immediate emergence from anesthesia, when nociceptive input and environmental disorientation are greatest. The later attenuation of pain differences may be explained by standardized postoperative analgesia, reassurance from PACU staff, and stabilization of the early postoperative environment. The absence of significant between-group differences in total 24-h analgesic consumption and pain at 24 h should not be interpreted as absence of an anxiety–pain relationship. Rather, it suggests that anxiety may be more strongly related to immediate postoperative pain perception and early recovery kinetics, while standardized analgesic protocols may reduce later group differences.^8^, ^11^, ^15^

Our study also extends the donor-specific literature in key ways. While living donor nephrectomy is increasingly safe and standardised under enhanced-recovery protocols, our results highlight that psychological readiness remains an overlooked determinant of efficiency and experience. A study emphasized the value of applying ERAS principles in living kidney donors; our data suggest that integrating preoperative anxiety screening may strengthen such pathways.^14^ Another study likewise identified pre-donation anxiety and limited pre-operative information as risk factors for worse donor quality of life, suggesting that psychological preparation has long-term relevance beyond immediate recovery.^3^

Clinically, these findings support incorporating brief, structured anxiety assessment into living donor perioperative pathways. Donors with elevated STAI-S scores may benefit from targeted counselling, expectation-setting, relaxation-based strategies, and improved perioperative communication. Such interventions may be particularly relevant in donor nephrectomy, where rapid recovery, pain control, and donor satisfaction are important quality indicators. Preoperative identification of high-anxiety donors may also help anesthesia and PACU teams anticipate recovery needs, optimize analgesic planning, and allocate postoperative resources more efficiently.^11^, ^14^

### Strengths and limitations of the study

The strengths of the present study lie in its prospective nature, the use of a widely used standardized anxiety measure with good internal consistency in this sample, which showed excellent internal consistency reliability (α = 0.875), a homogeneous group of donors treated according to standardized anesthesia protocols, and the measurement of several outcome variables of clinical importance.

However, limitations must be acknowledged. The single-centre design may limit generalisability to other centres or geographic regions. Because this study included medically cleared living kidney donors from a single transplant centre, the findings may not be directly generalizable to routine surgical patients with greater comorbidity burden. The sequential dependence among several recovery outcomes, limited perioperative confounder adjustment, categorization of STAI-S, and lack of reported regression diagnostics should also be considered. We did not measure trait anxiety, pain-catastrophising, coping style, or preoperative sleep quality, all potential moderators of anxiety-outcome relationships. Although our analgesic regimen was standardised, the narrow dose range may have restricted our capacity to detect analgesic-use differences across anxiety groups. Finally, the observational nature of the study precludes definitive causality; residual confounding by intraoperative or postoperative variables cannot be excluded. Although the sample size was adequate for the primary adjusted analysis, it was not designed to provide sufficient power for formal stratified subgroup analyses. Therefore, the interaction analyses by age and gender should be interpreted as exploratory and hypothesis-generating.

## Conclusions

Among living kidney donors undergoing donor nephrectomy under balanced general anesthesia, higher preoperative state anxiety scores were associated with lower early postoperative recovery scores. Higher anxiety was also associated with delayed extubation, longer Aldrete and PACU recovery times, greater early postoperative pain, prolonged hospital stay, and lower patient satisfaction. These findings suggest that routine preoperative anxiety assessment may help identify donors at risk of less favorable early postoperative recovery. However, larger multicentre studies with more rigorous adjustment for potential confounding factors are required to confirm these findings.

## CRediT authorship contribution statement

**Munawal Javaid:** Writing – review & editing, Writing – original draft, Investigation, Data curation. **Zargham Waheed:** Writing – review & editing, Writing – original draft, Methodology, Investigation. **Syed Jawad Ali Bukhari:** Writing – review & editing, Writing – original draft, Validation, Methodology, Formal analysis, Data curation, Conceptualization. **Maham Javed Chohan:** Writing – review & editing, Writing – original draft, Supervision, Methodology, Investigation, Formal analysis, Data curation, Conceptualization. **Amnah Khalid:** Writing – review & editing, Writing – original draft, Investigation, Formal analysis. **Sara Sarfraz:** Writing – review & editing, Writing – original draft, Visualization, Investigation, Formal analysis. **Zaeema Ahmad:** Writing – review & editing, Writing – original draft, Validation, Data curation. All authors have read and agreed to the published version of the manuscript.

## Consent for statement

Informed written consent was obtained from all participants before inclusion in the study.

## Ethical statement

The study was conducted in accordance with the ethical principles outlined in the Declaration of Helsinki. Ethical approval was obtained from the Institutional Ethical Review Committee (IERC) of Bahira International (SAFARI) Hospital, Rawalpindi, Pakistan (REF NO: IERC/BIH-RWP/2024/003) before the commencement of the study.

## Funding

This research received no specific grant from any funding agency in the public, commercial, or not-for-profit sectors.

## Data availability

The data that support the findings of this study are available from the corresponding author upon reasonable request.

## Declaration of competing interest

The authors declare that they have no known competing financial interests or personal relationships that could have appeared to influence the work reported in this paper.

## Footnotes

Appendix A Supplementary data associated with this article can be found in the online version at doi:10.1016/j.jatmed.2026.07.001.

## References

[1] Turksal E., Alper I., Sergin D. (2020). The effects of preoperative anxiety on anesthetic recovery and postoperative pain in patients undergoing donor nephrectomy. Braz J Anesthesiol.

[2] Galani V., Mazzola V., Prada P. (2024). Postoperative factors associated with psychological well-being of living kidney donors: results of a retrospective and qualitative study. Front Psychol.

[3] Menjivar A., Torres X., Manyalich M. (2020). Psychosocial risk factors for impaired health-related quality of life in living kidney donors: results from the ELIPSY prospective study. Sci Rep.

[4] Shebl M.A., Toraih E., Shebl M. (2025). Preoperative anxiety and its impact on surgical outcomes: a systematic review and meta-analysis. J Clin Transl Sci.

[5] Zeb U., Ahmed S., Shah L. (2024). Assessment of preoperative anxiety and the risk factors in patients booked for major elective general surgical procedures with amsterdam preoperative anxiety scale. J Popul Ther Clin Pharmacol.

[6] Chen M., Huang Y., Zhang J. (2024). Impact of preoperative anxiety on postoperative outcomes in patients undergoing minimally invasive thoracoscopic surgery: a prospective cohort study. Eur J Surg Oncol.

[7] Jaszczuk S., Natarajan S., Papalois V. (2022). Anaesthetic approach to enhanced recovery after surgery for kidney transplantation: a narrative review. J Clin Med.

[8] Han S., Park J., Hong S.H. (2020). Comparison of the impact of propofol versus sevoflurane on early postoperative recovery in living donors after laparoscopic donor nephrectomy: a prospective randomized controlled study. BMC Anesthesiol.

[9] Cho H.B., Kim M.G., Park S.Y. (2021). The influence of propofol-based total intravenous anesthesia on postoperative outcomes in end-stage renal disease patients: a retrospective observation study. PLOS ONE.

[10] Wu Q., Luo Y., Han M. (2023). The value of pain sensitivity questionnaire in predicting postoperative pain in living kidney donors: a prospective observational study. J Pain Res.

[11] Hou Y., Kang F., Liu H. (2023). Perioperative transcutaneous electrical acupoint stimulations as part of an enhanced recovery after surgery protocol for living donors undergoing nephrectomy: A randomized, controlled clinical trial. Heliyon.

[12] Kovač R., Juginović I., Delić N. (2024). The effect of epidural analgesia on quality of recovery (QoR) after open radical nephrectomy: randomized, prospective, and controlled trial. J Pers Med.

[13] Spielberger C.D. State-Trait Anxiety Inventory for Adults. Available from: 10.1037/t06496-000. [Accessed 1 November 2025].

[14] Byrne M.H.V., Mehmood A., Summers D.M. (2021). A systematic review of living kidney donor enhanced recovery after surgery. Clin Transplant.

[15] Stamenkovic D.M., Rancic N.K., Latas M.B. (2018). Preoperative anxiety and implications on postoperative recovery: what can we do to change our history. Minerva Anestesiol.

