# Supplementary material for: Association of preoperative state anxiety and early postoperative recovery in living kidney donors undergoing nephrectomy: A prospective observational study
Source: J Anesth Transl Med. 2026 Aug 11;5(3):171–8. doi: 10.1016/j.jatmed.2026.07.001 (PMC13482514; doi:10.1016/j.jatmed.2026.07.001)
Supplement: Supplementary file 1 [file mmc1.pdf]

Supplementary File S1. STROBE checklist for cohort studies

Manuscript title: Association of preoperative state anxiety and early postoperative recovery in living kidney donors undergoing nephrectomy: A prospective observational study

STROBE item no.	Recommendation	Reported in manuscript	Location in manuscript
1a	Indicate the study design with a commonly used term in the title or abstract.	Yes	Title, Abstract (Methods)
1b	Provide an informative and balanced summary of what was done and what was found.	Yes	Abstract
2	Explain the scientific background and rationale for the investigation being reported.	Yes	Introduction
3	State specific objectives, including any prespecified hypotheses.	Yes	Introduction (last paragraph)
4	Present key elements of study design early in the paper.	Yes	Methodology (Study design and approval)
5	Describe the setting, locations, and relevant dates, including periods of recruitment, exposure, follow-up, and data collection.	Yes	Methodology (Study design and approval; Data collection; Outcome measures)
6a	Give eligibility criteria, and sources and methods of selection of participants.	Yes	Methodology (Study participants)
6b	For matched studies, give matching criteria and number of exposed and unexposed.	Not applicable.	Not applicable
7	Clearly define all outcomes, exposures, predictors, potential confounders, and effect modifiers.	Yes	Methodology (Data collection; Outcome measures; Statistical analysis)
8	For each variable of interest, give sources of data and details of methods of assessment.	Yes	Methodology (Data collection; Outcome measures)
9	Describe any efforts to address potential sources of bias.	Yes	Methodology (Study participants; Anaesthesia protocols; Outcome measures; Statistical analysis)
10	Explain how the study size was arrived at.	Yes	Methodology (Sampling technique and sample size)
11	Explain how quantitative variables were handled in the analyses. If	Yes	Methodology (Statistical analysis)

	applicable, describe which groupings were chosen and why.		
12a	Describe all statistical methods, including those used to control for confounding.	Yes	Methodology (Statistical analysis)
12b	Describe any methods used to examine subgroups and interactions.	Yes	Methodology (Statistical analysis), Results (Interaction analysis)
12c	Explain how missing data were addressed.	Partly reported	Methodology (Ethical consideration/Data integrity; Statistical analysis)
12d	If applicable, explain how loss to follow-up was addressed.	Not applicable/minimal	Methodology (Outcome measures; Results)
12e	Describe any sensitivity analyses.	Partly addressed	Methodology (Statistical analysis; Limitations)
13a	Report the numbers of individuals at each stage of the study: eligible, included, followed up, and analysed.	Partly reported	Results (Baseline characteristics)
13b	Give reasons for non-participation at each stage.	Yes	Methodology (Study participants; Results)
13c	Consider use of a flow diagram.	No	NA
14a	Give characteristics of study participants and information on exposures and potential confounders.	Yes	Results (Table 1; Table 2; Supplementary Table S1)
14b	Indicate the number of participants with missing data for each variable of interest.	Yes	Results (Baseline characteristics and Statistical analysis)
14c	Summarize follow-up time.	Yes/Not applicable.	Methodology (Outcome measures; Results)
15	Report numbers of outcome events or summary measures over time.	Yes	Results (Table 3)
16a	Give unadjusted estimates and, if applicable, confounder-adjusted estimates and their precision.	Yes	Results (Table 4; Table 5)
16b	Report category boundaries when continuous variables are categorized.	Yes	Methodology (Statistical analysis), Results (Table 2)
16c	If relevant, consider translating estimates of relative risk into absolute risk for a meaningful time period.	Not applicable	Results (Table 5 paragraph)
17	Report other analyses done, including subgroup analyses,	Yes	Results (Interaction analysis; Supplementary Table S2)

	interactions, and sensitivity analyses.		
18	Summarize key results with reference to study objectives.	Yes	Discussion (first paragraph)
19	Discuss limitations, taking into account sources of potential bias or imprecision.	Yes	Strengths and limitations
20	Give a cautious overall interpretation considering objectives, limitations, multiplicity of analyses, similar studies, and other relevant evidence.	Yes	Discussion; Limitations; Conclusion
21	Discuss the generalisability/external validity of the study results.	Yes	Discussion; Limitations
22	Give the source of funding and the role of funders.	Yes	Funding statement
