# Supplementary material for: Association of preoperative state anxiety and early postoperative recovery in living kidney donors undergoing nephrectomy: A prospective observational study
Source: J Anesth Transl Med. 2026 Aug 11;5(3):171–8. doi: 10.1016/j.jatmed.2026.07.001 (PMC13482514; doi:10.1016/j.jatmed.2026.07.001)
Supplement: Supplementary file 2 [file mmc2.pdf]

Supplementary Table S1. Baseline characteristics across anxiety categories

Variable	Subgroup	Low anxiety	Moderate anxiety	High anxiety	Test statistic	p value
Age	Mean ± SD	38.00 ± 5.34	34.76 ± 6.77	36.52 ± 7.53	H = 4.075	0.130
Body mass index	Mean ± SD	24.78 ± 4.69	22.58 ± 3.85	22.46 ± 4.78	H = 5.922	0.052
Gender	Male, n (%)	19 (43.2)	13 (39.4)	9 (39.1)	$\chi^2 = 0.155$	0.925
	Female, n (%)	25 (56.8)	20 (60.6)	14 (60.9)		
Marital Status	Unmarried, n (%)	6 (13.6)	14 (42.4)	7 (30.4)	$\chi^2 = 8.108$	**0.017**
	Married, n (%)	38 (86.4)	19 (57.6)	16 (69.6)		
ASA Physical Status	ASA I, n (%)	36 (81.8)	25 (75.8)	16 (69.6)	$\chi^2 = 1.323$	0.516
	ASA II, n (%)	8 (18.2)	8 (24.2)	7 (30.4)		
Educational Level	Primary, n (%)	9 (20.5)	7 (21.2)	5 (21.7)	$\chi^2 = 6.308$	0.390
	Secondary, n (%)	16 (36.4)	11 (33.3)	12 (52.2)		
	Graduate, n (%)	13 (29.5)	8 (24.2)	6 (26.1)		
	Postgraduate, n (%)	6 (13.6)	7 (21.2)	0 (0.0)		
Employment Status	Employed, n (%)	11 (25.0)	6 (18.2)	5 (21.7)	$\chi^2 = 3.070$	0.800
	Unemployed, n (%)	15 (34.1)	8 (24.2)	8 (34.8)		
	Housewife, n (%)	7 (15.9)	10 (30.3)	5 (21.7)		
	Student, n (%)	11 (25.0)	9 (27.3)	5 (21.7)		
History of previous Surgery	Yes, n (%)	6 (13.6)	5 (15.2)	2 (8.7)	$\chi^2 = 0.528$	0.768
	No, n (%)	38 (86.4)	28 (84.8)	21 (91.3)		
Smoking History	Yes, n (%)	4 (9.1)	3 (9.1)	2 (8.7)	$\chi^2 = 0.003$	0.998
	No, n (%)	40 (90.9)	30 (90.9)	21 (91.3)		

Legend: Data are presented as n (%) unless otherwise stated. Age and body mass index were compared using the Kruskal–Wallis test. Categorical variables were compared using the chi-square test. Percentages are calculated within anxiety categories. H, Kruskal–Wallis test statistic; χ^2, chi-square statistic; ASA, American Society of Anesthesiologists.

Note: Marital status differed significantly across anxiety categories and was therefore included as an additional covariate in the multivariable regression model.

Supplementary Table S2. Exploratory interaction analyses for PACU stay duration

**Interaction term**	**B**	**SE**	**Standardized β**	**p value**
STAI-S × age	0.000	0.010	0.004	0.961
STAI-S × gender	-0.169	0.135	-0.143	0.215

Legend: Interaction terms were entered separately into the adjusted regression model to avoid overfitting. The dependent variable was total PACU stay duration.
